# Numerical solution of the one-dimensional fractional convection diffusion equations based on Chebyshev operational matrix

**DOI:** 10.1186/s40064-016-2832-y

**Published:** 2016-07-22

**Authors:** Jiaquan Xie, Qingxue Huang, Xia Yang

**Affiliations:** College of Mechanical Engineering, Taiyuan University of Science and Technology, Taiyuan, 030024 Shanxi China

**Keywords:** Chebyshev operational matrix, One-dimensional fractional convection diffusion equations, Numerical solution, Variable coefficients, Tau method

## Abstract

In this paper, we are concerned with nonlinear one-dimensional fractional convection diffusion equations. An effective approach based on Chebyshev operational matrix is constructed to obtain the numerical solution of fractional convection diffusion equations with variable coefficients. The principal characteristic of the approach is the new orthogonal functions based on Chebyshev polynomials to the fractional calculus. The corresponding fractional differential operational matrix is derived. Then the matrix with the Tau method is utilized to transform the solution of this problem into the solution of a system of linear algebraic equations. By solving the linear algebraic equations, the numerical solution is obtained. The approach is tested via examples. It is shown that the proposed algorithm yields better results. Finally, error analysis shows that the algorithm is convergent.

## Background

Convection diffusion equations are regarded as a kind of basic equations of motion, which have been applied in describing water flow movement (Hu et al. 2016; Colla et al. 2015; Su 2014), material transport and diffusion (Liu et al. 2016; Calo et al. 2015; Karalashvili et al. 2015; Fang and Deng 2014). Convection diffusion equations are widely used in water conservancy project (Hu et al. 2016; Su 2014), environmental engineering and aviation (Hernandez et al. 1995), Marine (Farahani et al. 2015), chemical (Colla et al. 2015; Diehl 2015), metallurgy (Zaib and Shafile 2014), so the study of numerical solutions of convection diffusion equations has important theoretical and practical significance.

There are many numerical methods for solving convection diffusion problems, such as finite difference methods (Kaya 2015), finite element methods (He et al. 2015; Mudunuru and Nakshatrala 2016; Wu et al. 2013), wavelet methods (Zhou and Xu 2014, 2016; Chen et al. 2010; EI-Gamel 2006), polynomials methods (Li et al. 2016), iterative methods (Das and Mehrmann 2015; Das 2015; Das and Natesan 2014). Operational matrix methods are new on this area which have made great success presently. Especially in recent 20 years, the articles about operational matrix methods are springing up. In Ref. Abbasbandy et al. (2015), the authors proposed the operational matrix method of fractional-order Legendre functions for solving time-fractional convection–diffusion equations.

**Fig. 1:**
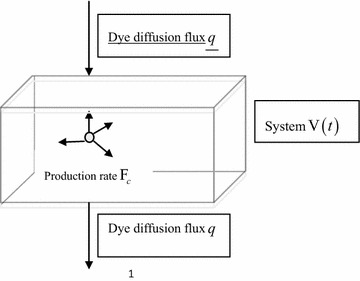
The schematic diagram of dye diffusion in the system

In the following we mention some real-world applications of convection diffusion equations. In this paper, we take an example of a diffusion kinetic model, the flow rate is ***u*** = *u*(*x*, *t*), dye concentration is *c*(*x*, *t*) and diffusion flux of dyes is ***q*** = *q*(*x*, *t*). We also set up a variety of biological, chemical and other factors to control the production rate of dye (Production of unit volume per unit time) *F*_*c*_. In the flow field, a system (its volume can be arbitrary), as shown in Fig. 1, is obtained. The space occupied by the system during the flow of *V*(*t*) is controlled at the time *t*.

According to the model, and the related initial-boundary conditions, we obtain the one-dimensional convection diffusion equations (Chen and Jin (2007)).

1 $$\left\{ {\begin{array}{*{20}l} {\frac{\partial c}{\partial t} + u\frac{\partial c}{\partial x} = D\frac{{\partial^{2} c}}{{\partial x^{2} }},} &\quad {t > 0, \, 0 < x < l,} \\ {c\left( {x,0} \right) = 0,} &\quad {0 < x < l,} \\ {c\left( {0,t} \right) = C_{0} ,} &\quad {c\left( {l,t} \right) = 0,t > 0,} \hfill \\ \end{array} } \right.$$

In this paper, a numerical approach based on Chebyshev operational matrix is proposed for solving one-dimensional fractional convection diffusion equations with variable coefficients of the following form:

2 $$\left\{ {\begin{array}{*{20}l} {\frac{\partial u}{\partial t} + a\left( x \right)\frac{\partial u}{\partial x} = b\left( x \right)\frac{{\partial^{\nu } u}}{{\partial x^{\nu } }} + g\left( {x,t} \right),} &\quad {0 < x < 1,\,t > 0,} \hfill \\ {u(x,0) = h_{1} \left( x \right),} &\quad {0 < x < 1,} \hfill \\ {u\left( {0,t} \right) = g_{1} \left( t \right),u\left( {1,t} \right) = g_{2} \left( t \right),} &\quad {t > 0,} \hfill \\ \end{array} } \right.$$

the parameter *ν* refers to the fractional order of spatial derivative with 1 < *ν*≤2. In the proposed method, the operational matrices of fractional-order are employed to obtain the numerical solutions of Eq. (2).

The paper is organized as follows: In “Preliminaries and notations” section, some basic definitions and mathematical preliminaries of fractional calculus are introduced. The fractional differential operational matrix is given in “The fractional derivative operational matrix *P*^(ν)^” section. We mainly illustrate the proposed method in “Description of the proposed method” section. In “Error analysis” section, the convergence of the proposed approach is proved. In “Numerical simulation” section, the proposed approach is applied to test several numerical examples. Finally, a conclusion is given in “Conclusion” section.

## Preliminaries and notations

### The basic definitions of fractional integral and differential operator

#### **Definition 1**

The Riemann–Liouville fractional integral operator *I*^*ν*^ of order *ν* is defined as

3 $$\left( {I^{\nu } f} \right)\left( t \right) = \left\{ \begin{array}{ll} \frac{1}{\varGamma \left( \nu \right)}\int_{\;0}^{\;t} {\left( {t - \tau } \right)^{\nu - 1} } f\left( \tau \right)\;d\tau ,&\quad \nu > 0; \\ f\left( t \right),&\quad \nu = 0. \end{array} \right.$$

#### **Definition 2**

The Riemann–Liouville fractional differential operator *D*^*ν*^ of order *ν* is defined as

4 $$D^{\nu } f\left( t \right) = \left\{ \begin{array}{ll} \frac{1}{{\varGamma \left( {m - \nu } \right)}}\frac{{{\text{d}}^{m} }}{{{\text{d}}t^{m} }}\int_{0}^{t} {\frac{f\left( s \right)}{{\left( {t - s} \right)^{\nu - m + 1} }}ds}, &\quad\nu > 0,m - 1 \le \nu < m; \\ \frac{{{\text{d}}^{m} f\left( t \right)}}{{{\text{d}}t^{m} }},&\quad \nu = m. \\ \end{array} \right.$$

#### **Definition 3**

The Caputo fractional differential operator is defined as

5 $$D^{\nu } f\left( t \right) = \left\{ {\begin{array}{*{20}l} {\frac{1}{{\varGamma \left( {m - \nu } \right)}}\int_{0}^{t} {\frac{{f^{\left( m \right)} \left( \tau \right)}}{{\left( {t - \tau } \right)^{\nu - m + 1} }}} d\tau ,} &\quad {m - 1 \le \nu < m;} \\ {\frac{{{\text{d}}^{m} f\left( t \right)}}{{{\text{d}}t^{m} }},} &\quad {\nu = m.} \\ \end{array} } \right.$$

For the Caputo derivative, we have

6 $$D^{\nu } t^{\gamma } = \left\{ {\begin{array}{*{20}l} {0,} &\quad {{\text{for}}\,\gamma \in N_{0} \,{\text{and}}\,{\kern 1pt} \gamma < \left\lceil \nu \right\rceil ;} \hfill \\ {\frac{{\varGamma \left( {\gamma + 1} \right)}}{{\varGamma \left( {\gamma + 1 - \nu } \right)}}t^{\gamma - \nu } ,} &\quad {{\text{for}}\,\gamma \in N_{0} \,{\text{and}}{\kern 1pt} \,\gamma \ge \left\lceil \nu \right\rceil \,{\text{or}}\,\gamma \notin N_{0} \,{\text{and}}\,\gamma > \left\lfloor \nu \right\rfloor .} \\ \end{array} } \right.$$

### Properties of the Chebyshev polynomials

The well-known Chebyshev polynomials are defined on the interval (−1, 1) and can be determined with the aid of the following recurrence formula (Doha et al. 2011):

$$T_{i + 1} \left( t \right) = 2tT_{i} \left( t \right) - T_{i - 1} \left( t \right),{\kern 1pt} \quad i = 1,2, \ldots$$

where *T*_0_(*t*) = 1 and *T*_1_(*t*) = *t*. In order to use these polynomials on the interval *x* ∈ (0, 1), we define the Chebyshev polynomials by introducing the change of variable *t* = 2*x* − 1. Let the Chebyshev polynomials *T*_*i*_(*t*) = 2*x* − 1 are denoted by *T*_*i*_(*x*), then *T*_*i*_(*x*) can be obtained as follows:

7 $$T_{i + 1} \left( x \right) = 2\left( {2x - 1} \right)T_{i} \left( x \right) - T_{i - 1} \left( x \right),{\kern 1pt} {\kern 1pt} {\kern 1pt} {\kern 1pt} {\kern 1pt} i = 1,2, \ldots$$

where *T*_0_(*x*) = 1 and *T*_1_(*x*) = 2*x* − 1. The analytic form of the Chebyshev polynomials *T*_*i*_(*x*) of degree *i* is given by

8 $$T_{i} \left( x \right) = i\sum\limits_{k = 0}^{i} {( - 1)^{i - k} \frac{{\left( {i + k - 1} \right)!2^{2k} }}{{\left( {i - k} \right)!\left( {2k} \right)!}}} x^{k} ,$$

where *T*_*i*_(0) = (−1)^*i*^ and *T*_*i*_(1) = 1.

The orthogonally condition is

9 $$\int_{0}^{1} {T_{j} } \left( x \right)T_{k} \left( x \right)w\left( x \right)dx = h_{k} ,$$

where the weight function $w\left( x \right) = \frac{1}{{\sqrt {x - x^{2} } }}$ and $$h_{k} = \left\{ {\begin{array}{*{20}l} {\frac{{b_{k} }}{2}\pi ,} \hfill & {k = j,} \hfill \\ {0,} \hfill & {k \ne j,} \hfill \\ \end{array} } \right.\quad b_{0} = 2,\;b_{k} = 1,\;k \ge 1.$$

### Function approximation

Suppose $u\left( x \right) \in L^{2} \left( {0,1} \right),$ it may be expressed in terms of the Chebyshev polynomials as

10 $$u\left( x \right) = \sum\limits_{i = 0}^{\infty } {c_{i} T_{i} \left( x \right)} ,$$

where the coefficients *c*_*i*_ is given by

11 $$c_{i} = \frac{1}{{h_{i} }}\int_{0}^{1} {u\left( x \right)T_{i} } \left( x \right)w\left( x \right)dx,\quad i = 0,1,2, \ldots$$

If we consider truncated series in Eq. (10), then we have:

12 $$u\left( x \right) \simeq u_{M} \left( x \right) = \sum\limits_{i = 0}^{M} {c_{i} } T_{i} \left( x \right) = \varvec{C}^{T}\varvec{\varPhi}\left( x \right),$$

where

13 $$\varvec{C} = \left[ {c_{0} ,c_{1} , \ldots ,c_{M} } \right]^{T} ;{\kern 1pt} {\kern 1pt} {\kern 1pt} {\kern 1pt} {\kern 1pt} {\kern 1pt} {\kern 1pt} {\kern 1pt} {\kern 1pt} {\kern 1pt}\varvec{\varPhi}\left( x \right) = \left[ {T_{0} \left( x \right),T_{1} \left( x \right), \ldots ,T_{M} \left( x \right)} \right]^{T} .$$

Then the derivative of vector $\varvec{\varPhi}$ can be expressed by

14 $$\frac{{{\text{d}}\varvec{\varPhi}\left( x \right)}}{{{\text{d}}x}} = \varvec{P}^{\left( 1 \right)}\varvec{\varPhi}\left( x \right),$$

where *P*^(1)^ is the (*M* + 1) × (*M* + 1) operational matrix of derivative given by

15 $$\varvec{P}^{\left( 1 \right)} = \left( {p_{ij} } \right) = \left\{ {\begin{array}{*{20}l} {\frac{4i}{{b_{j} }},} \hfill & {j = 0,1, \ldots ,\,i = j + k,\;\left\{ {\begin{array}{*{20}l} {k = 1,3,5, \ldots ,M,} \hfill & {{\text{if}}\,M\,{\text{is}}\,{\text{odd,}}} \hfill \\ {k = 1,3,5, \ldots ,M - 1,} \hfill & {{\kern 1pt} {\text{if}}\,M\,{\text{is}}\,{\text{even,}}} \hfill \\ \end{array} } \right.} \hfill \\ {0,} \hfill & {otherwise} \hfill \\ \end{array} } \right.$$

For example for even *M*, we have

$$\varvec{P}^{\left( 1 \right)} = 2 \times \left( {\begin{array}{*{20}c} 0 & 0 & 0 & 0 & 0 & \cdots & 0 & 0 \\ 1 & 0 & 0 & 0 & 0 & \cdots & 0 & 0 \\ 0 & 4 & 0 & 0 & 0 & \cdots & 0 & 0 \\ 3 & 0 & 6 & 0 & 0 & \cdots & 0 & 0 \\ 0 & 8 & 0 & 8 & 0 & \cdots & 0 & 0 \\ 5 & 0 & {10} & 0 & {10} & \cdots & 0 & 0 \\ \vdots & \vdots & \vdots & \vdots & \vdots & \ldots & \vdots & \vdots \\ {M - 1} & 0 & {2M - 2} & 0 & {2M - 2} & \cdots & 0 & 0 \\ 0 & {2M} & 0 & {2M} & 0 & \cdots & {2M} & 0 \\ \end{array} } \right)$$

Based on the function approximation theory which the solution function is expressed as orthogonal polynomials. For arbitrary function $u\left( {x,t} \right) \in L^{2} \left( {\left( {0,1} \right) \times \left( {0,1} \right)} \right)$, it can be expanded as the following way

16 $$u\left( {x,t} \right) = \sum\limits_{i = 0}^{\infty } {\sum\limits_{j = 0}^{\infty } {u_{ij} T_{i} } } \left( x \right)T_{j} \left( t \right),$$

where

17 $$u_{ij} = \frac{1}{{h_{i} h_{j} }}\int_{0}^{1} {\int_{0}^{1} {u\left( {x,t} \right)T_{i} \left( x \right)T_{j} \left( t \right)} } w\left( x \right)w\left( t \right)dxdt,\quad i,j = 0,1,2, \ldots$$

If we consider truncated series in Eq. (16), then we have:

18 $$u\left( {x,t} \right) \approx \sum\limits_{i = 0}^{M} {\sum\limits_{j = 0}^{N} {u_{ij} T_{i} } } \left( x \right)T_{j} \left( t \right) =\varvec{\varPhi}\left( x \right)^{T} \varvec{U\varPhi }\left( t \right),$$

where

19 $$\varvec{\varPhi}\left( x \right) = \left[ {T_{0} \left( x \right),T_{1} \left( x \right), \ldots ,T_{M} \left( x \right)} \right]^{T} ;\varvec{\varPhi}\left( t \right) = \left[ {T_{0} \left( t \right),T_{1} \left( t \right), \ldots ,T_{N} \left( t \right)} \right]^{T} ;\varvec{U} = \left\{ {u_{ij} } \right\}_{i,j = 0}^{M,N} .$$

In this paper, we use the Tau method (Bhrawy et al. 2011; Dehghan and Saadatmandi 2006) to compute the coefficients *u*_*ij*_.

## The fractional derivative operational matrix *P*^(ν)^

The main objective of this section is to prove the following theorem for the fractional derivatives of the Chebyshev polynomials.

### **Lemma 1**

*Let T*_*i*_(*x*) *be a Chebyshev polynomial; then* (Doha et al. 2011)

20 $$D^{\nu } T_{i} \left( x \right) = 0,\quad i = 0,1,2, \ldots ,\left\lceil \nu \right\rceil - 1, \quad \nu > 0.$$

### *Proof*

This Lemma can be easily proved by making use of relation (5) and (7).

### **Theorem 1**

*Let*$\varvec{\varPhi}\left( x \right)$*be the Chebyshev vector defined in Eq.* (12) *and suppose*$\nu > 0$*; then*

21 $$D^{\nu }\varvec{\varPhi}\left( x \right) \simeq \varvec{P}^{\left( \nu \right)}\varvec{\varPhi}\left( x \right),$$

where *P*^(ν)^is the (*M* + 1) × (*M* + 1) differential operational matrix of order ν in the Caputo sense and it is defined as follows:

$$\varvec{P}^{\left( \nu \right)} = \left( {\begin{array}{*{20}c} 0 & 0 & 0 & \cdots & 0 \\ \vdots & \vdots & \vdots & \cdots & \vdots \\ 0 & 0 & 0 & \cdots & 0 \\ {S_{\nu } \left( {\left\lceil \nu \right\rceil ,0} \right)} & {S_{\nu } \left( {\left\lceil \nu \right\rceil ,1} \right)} & {S_{\nu } \left( {\left\lceil \nu \right\rceil ,2} \right)} & \cdots & {S_{\nu } \left( {\left\lceil \nu \right\rceil ,M} \right)} \\ \vdots & \vdots & \vdots & \cdots & \vdots \\ {S_{\nu } \left( {i,0} \right)} & {S_{\nu } \left( {i,1} \right)} & {S_{\nu } \left( {i,2} \right)} & \cdots & {S_{\nu } \left( {i,M} \right)} \\ \vdots & \vdots & \vdots & \cdots & \vdots \\ {S_{\nu } \left( {M,0} \right)} & {S_{\nu } \left( {M,1} \right)} & {S_{\nu } \left( {M,2} \right)} & \cdots & {S_{\nu } \left( {M,M} \right)} \\ \end{array} } \right)$$

where

$$S_{\nu } \left( {i,j} \right) = \sum\limits_{k = \left\lceil \nu \right\rceil }^{i} {\frac{{\left( { - 1} \right)^{i - k} 2i\left( {i + k - 1} \right)!\varGamma \left( {k - \nu + \frac{1}{2}} \right)}}{{b_{j} \varGamma \left( {k + \frac{1}{2}} \right)\left( {i - k} \right)!\varGamma \left( {k - \nu - j + 1} \right)\varGamma \left( {k - \nu + j + 1} \right)}}} .\quad i = \left\lceil \nu \right\rceil ,\left\lceil \nu \right\rceil + 1, \ldots ,M.$$

### *Proof*

The analytical form of the Chebyshev polynomials *T*_*i*_(*x*) of degree *i* is given by Eq. (8), using Eqs. (6) and (8) we have

22 $$\begin{aligned} D^{\nu } T_{i} \left( x \right) & = i\sum\limits_{k = 0}^{i} {\left( { - 1} \right)^{i - k} \frac{{\left( {i + k - 1} \right)!2^{2k} }}{{\left( {i - k} \right)!\left( {2k} \right)!}}} D^{\nu } x^{k} \\ & = i\sum\limits_{k = 0}^{i} {\left( { - 1} \right)^{i - k} } \frac{{\left( {i + k - 1} \right)!2^{2k} k!}}{{\left( {i - k} \right)!\left( {2k} \right)!\varGamma \left( {k + \nu + 1} \right)}}x^{k - \nu } ,\quad i = \left\lceil \nu \right\rceil ,\left\lceil \nu \right\rceil + 1, \ldots ,M. \\ \end{aligned}$$

Now, approximate $x^{k - \nu }$ by (*M* + 1) terms of the Chebyshev series, we have

23 $$x^{k - \nu } = \sum\limits_{j = 0}^{M - 1} {c_{kj} } T_{j} \left( x \right),$$

where *c*_*kj*_ is given from Eq. (11) with $u\left( x \right) = x^{k - \nu }$, and

24 $$c_{kj} = \left\{ {\begin{array}{*{20}l} {\frac{1}{\sqrt \pi }\frac{{\varGamma \left( {k - \nu + \frac{1}{2}} \right)}}{{\varGamma \left( {k - \nu + 1} \right)}},} &\quad {j = 0,} \\ {\frac{j}{\sqrt \pi }\sum\limits_{r = 0}^{j} {\left( { - 1} \right)^{j - r} \frac{{\left( {j + r - 1} \right)!2^{2r + 1} \varGamma \left( {k + r - \nu + \frac{1}{2}} \right)}}{{\left( {j - r} \right)!\left( {2r} \right)!\varGamma \left( {k + r - \nu + 1} \right)}},} } &\quad {j = 1,2, \ldots M.} \\ \end{array} } \right.$$

Employing Eqs. (22)–(24) we get

25 $$D^{\nu } T_{i} \left( x \right) = \sum\limits_{j = 0}^{M} {S_{\nu } } \left( {i,j} \right)T_{j} \left( x \right),\quad i = \left\lceil \nu \right\rceil ,\left\lceil \nu \right\rceil + 1, \ldots M.$$

where $$S_{\nu } \left( {i,j} \right) = \sum\nolimits_{k = \left\lceil \nu \right\rceil }^{i} {\theta_{ijk} }$$, and

$$\begin{aligned} \theta_{ijk} = \left\{ {\begin{array}{*{20}l} {\frac{{i\left( { - 1} \right)^{i - k} \left( {i + k - 1} \right)!2^{2k} k!\varGamma \left( {k - \nu + \frac{1}{2}} \right)}}{{\left( {i - k} \right)!\left( {2k} \right)!\sqrt \pi \left( {\varGamma \left( {k - \nu + 1} \right)} \right)^{2} }},} \hfill & {j = 0,} \hfill \\ {\frac{{\left( { - 1} \right)^{i - k} ij\left( {i + k - 1} \right)!2^{2k + 1} k!}}{{\left( {i - k} \right)!\left( {2k} \right)!\varGamma \left( {k - \nu + 1} \right)\sqrt \pi }}} \hfill & {} \hfill \\ {\times \sum\limits_{r = 0}^{j} {\frac{{\left( { - 1} \right)^{j - r} \left( {j + r - 1} \right)!2^{2r} \varGamma \left( {k + r - \nu + \frac{1}{2}} \right)}}{{\left( {j - r} \right)!\left( {2r} \right)!\varGamma \left( {k + r - \nu + 1} \right)}},{\kern 1pt} } } \hfill & {j = 1,2, \ldots M.} \hfill \\ \end{array} } \right. \hfill \\ \hfill \\ \end{aligned}$$

After some lengthy manipulation, $\theta_{i,j,k}$ may be put in the form

26 $$\theta_{ijk} = \frac{{\left( { - 1} \right)^{i - k} 2i\left( {i + k - 1} \right)!\varGamma \left( {k - \nu + \frac{1}{2}} \right)}}{{b_{j} \varGamma \left( {k + \frac{1}{2}} \right)\left( {i - k} \right)!\varGamma \left( {k - \nu - j + 1} \right)\varGamma \left( {k - \nu + j + 1} \right)}},{\kern 1pt} \quad j = 0,1, \ldots M.$$

where $$b_{0} = 2,b_{j} = 1,j \ge 1$$.

Accordingly, Eq. (21) can be written in a vector form as follows:

27 $$D^{\nu } T_{i} \left( x \right) \simeq \left[ {S_{\nu } \left( {i,0} \right),S_{\nu } \left( {i,1} \right),S_{\nu } \left( {i,2} \right), \ldots S_{\nu } \left( {i,M} \right)} \right]\varvec{\varPhi}\left( x \right),{\quad}i = \left\lceil \nu \right\rceil ,\left\lceil \nu \right\rceil + 1, \ldots ,M.$$

Also, according to Lemma 1, we can write

28 $$D^{\nu } T_{i} \left( x \right) \simeq \left[ {0,0,0, \ldots ,0} \right]\varvec{\varPhi}\left( x \right),\quad i = 0,1, \ldots ,\left\lceil \nu \right\rceil - 1.$$

A combination of Eqs. (27) and (28) leads to the desired result.

## Description of the proposed method

In the section, we will use the Chebyshev polynomials operational matrix of fractional derivative to obtain the numerical solutions of one-dimensional fractional convection diffusion equations with variable coefficients.

Here, for simplicity we consider the convection diffusion equations of the following form:

29 $$\frac{\partial u}{\partial t} + a\left( x \right)\frac{\partial u}{\partial x} = b\left( x \right)\frac{{\partial^{\nu } u}}{{\partial x^{\nu } }} + f\left( {x,t} \right),\quad 1 < \nu \le 2.$$

where ${{\partial^{\nu } } \mathord{\left/ {\vphantom {{\partial^{\nu } } {\partial x^{\nu } }}} \right. \kern-0pt} {\partial x^{\nu } }}$ denote fractional derivatives in the Caputo’s sense, with the initial-boundary conditions

30 $$\begin{aligned} u\left( {x,0} \right) & = h_{1} \left( x \right),\quad 0 < x < 1, \\ u\left( {0,t} \right) & = g_{1} \left( x \right),\,u\left( {1,t} \right) = g_{2} \left( t \right),\quad t > 0. \\ \end{aligned}$$

In order to use the Chebyshev polynomials, we first approximate

31 $$u\left( {x,t} \right) \approx\varvec{\varPhi}^{\rm T} \left( x \right)\varvec{U\varPhi }\left( t \right),$$

where $\varvec{U} = \left[ {u_{ij} } \right]_{{\left( {M + 1} \right) \times \left( {N + 1} \right)}}$ is an unknown matrix.

The following is the product of two vectors based on Chebyshev operational matrix method. Let (Bhrawy et al. 2015)

32 $$\varvec{\varPhi}\left( x \right)\varvec{\varPhi}^{T} \left( x \right)\varvec{C} \approx \tilde{\varvec{Q}}^{T}\varvec{\varPhi}\left( x \right),$$

where $\tilde{\varvec{Q}}$ is the (*M* + 1) × (*M* + 1) operational matrix with the element $\tilde{\varvec{q}}_{kj}$. In virtue of Eq. (12) and above relation, enable us to write

33 $$\sum\limits_{i = 0}^{M} {c_{i} } T_{i} \left( x \right)T_{j} \left( x \right) = \sum\limits_{i = 0}^{M} {\tilde{\varvec{q}}_{ij} } T_{i} \left( x \right),\quad j = 0,1,2, \ldots ,M.$$

Multiplying both sides of the above equation by $T_{k} \left( x \right)\omega \left( x \right),{\kern 1pt} k = 0,1, \ldots ,M$ and integrating the result from 0 to 1, we obtain

34 $$\sum\limits_{i = 0}^{M} {c_{i} } \int_{0}^{1} {T_{i} } \left( x \right)T_{j} \left( x \right)T_{k} \left( x \right)\omega \left( x \right)dx = \tilde{\varvec{q}}_{kj} \int_{0}^{1} {T_{k} } \left( x \right)T_{k} \left( x \right)\omega \left( x \right)dx$$

Equation (34) yields

35 $$\tilde{\varvec{q}}_{kj} = \frac{1}{{h_{k} }}\sum\limits_{i = 0}^{M} {\left( {q_{i} \int_{0}^{1} {T_{i} \left( x \right)T_{j} \left( x \right)T_{k} \left( x \right)\omega \left( x \right)} } \right)} ,\quad k,j = 0,1, \ldots ,M.$$

Now, using Eqs. (12), (14), (21) and (32), we obtain

36 $$\frac{\partial u}{\partial t} \approx\varvec{\varPhi}^{T} \left( x \right)\varvec{UP}^{\left( 1 \right)}\varvec{\varPhi}\left( t \right),$$

37 $$a\left( x \right)\frac{\partial u}{\partial x} \approx A^{T}\varvec{\varPhi}\left( x \right)\varvec{\varPhi}^{T} \left( x \right)\left( {\varvec{P}^{\left( 1 \right)} } \right)^{T} \varvec{U\varPhi }\left( t \right) \approx\varvec{\varPhi}^{T} \left( x \right)\tilde{\varvec{A}}^{T} \left( {\varvec{P}^{\left( 1 \right)} } \right)^{T} \varvec{U\varPhi }\left( t \right),$$

and

38 $$b\left( x \right)\frac{{\partial^{\nu } u}}{{\partial x^{\nu } }} \approx \varvec{B}^{T}\varvec{\varPhi}\left( x \right)\varvec{\varPhi}^{T} \left( x \right)\left( {\varvec{P}^{v} } \right)^{T} \varvec{U\varPhi }\left( t \right) \approx\varvec{\varPhi}^{T} \left( x \right)\tilde{\varvec{B}}^{T} \left( {\varvec{P}^{v} } \right)^{T} \varvec{U\varPhi }\left( t \right),$$

Also, using Eq. (18), the function *g*(*x*, *t*) in Eq. (29) can be approximated as

39 $$f\left( {x,t} \right) \approx\varvec{\varPhi}^{T} \left( x \right)\varvec{F\varPhi }\left( t \right),$$

where $\varvec{F} = \left[ {f_{ij} } \right]$ is a (*M* + 1) × (*N* + 1) known matrix. Substituting Eqs. (31)–(39) in Eq. (29) yields

40 $$\varvec{\varPhi}^{T} \left( x \right)\varvec{UP}^{\left( 1 \right)}\varvec{\varPhi}\left( t \right) +\varvec{\varPhi}^{T} \left( x \right)\tilde{\varvec{A}}^{T} \left( {\varvec{P}^{\left( 1 \right)} } \right)^{T} \varvec{U\varPhi }\left( t \right) \approx\varvec{\varPhi}^{T} \left( x \right)\tilde{\varvec{B}}^{T} \left( {\varvec{P}^{v} } \right)^{T} \varvec{U\varPhi }\left( t \right) +\varvec{\varPhi}^{T} \left( x \right)\varvec{G\varPhi }\left( t \right)$$

The entries of vector $\varvec{\varPhi}\left( x \right)$ and $\varvec{\varPhi}\left( t \right)$ in Eq. (40) are independent, so we get

41 $$H = \varvec{UP}^{\left( 1 \right)} + \tilde{\varvec{A}}^{T} \left( {\varvec{P}^{\left( 1 \right)} } \right)^{T} \varvec{U} - \tilde{\varvec{B}}^{T} \left( {\varvec{P}^{v} } \right)^{T} \varvec{U} - \varvec{F} \approx 0$$

Here, we choose *MN* − *N* equations of Eq. (41) as

42 $$H_{ij} \approx 0,\quad \left( {i = 2,3, \ldots ,M,j = 1,2, \ldots ,N} \right)$$

We can also approximate the function *h*_1_(*x*), *g*_1_(*t*) and *g*_2_(*t*) as

43 $$h_{1} \left( x \right) \approx\varvec{\varPhi}^{T} \left( x \right)\varvec{H}_{1} ,{\kern 1pt} {\kern 1pt} {\kern 1pt} {\kern 1pt} {\kern 1pt} g_{1} \left( t \right) \approx \varvec{G}_{1}^{T}\varvec{\varPhi}\left( t \right),{\kern 1pt} {\kern 1pt} {\kern 1pt} {\kern 1pt} g_{2} \left( t \right) \approx \varvec{G}_{2}^{T}\varvec{\varPhi}\left( t \right)$$

where ***H***_1_, ***G***_1_, ***G***_2_ are known vectors.

Applying Eqs. (31) and (44) in the boundary conditions Eq. (30), we get

44 $$\begin{aligned}&\varvec{\varPhi}^{T} \left( x \right)\varvec{U\varPhi }\left( 0 \right) \approx\varvec{\varPhi}^{T} \left( x \right)\varvec{H}_{1} ,\\ &\varvec{\varPhi}^{T} \left( 0 \right)\varvec{U\varPhi }\left( t \right) \approx \varvec{G}_{1}^{T}\varvec{\varPhi}\left( t \right),\varvec{\varPhi}^{T} \left( 1 \right)\varvec{U\varPhi }\left( t \right) \approx \varvec{G}_{2}^{T}\varvec{\varPhi}\left( t \right), \\ \end{aligned}$$

The entries of vector $\varvec{\varPhi}\left( x \right)$ and $\varvec{\varPhi}\left( t \right)$ are independent, so from Eq. (44) we can obtain

45 $$\varLambda_{1} = \varvec{U\varPhi }\left( 0 \right) - \varvec{H}_{1} \approx 0,{\kern 1pt} {\kern 1pt} {\kern 1pt} {\kern 1pt} {\kern 1pt} \varLambda_{2} =\varvec{\varPhi}^{T} \left( 0 \right)\varvec{U} - \varvec{G}_{1}^{T} \approx 0,{\kern 1pt} {\kern 1pt} {\kern 1pt} {\kern 1pt} \varLambda_{3} =\varvec{\varPhi}^{T} \left( 1 \right)\varvec{U} - \varvec{G}_{2}^{T} \approx 0,$$

By choosing the (*M* − 1) equations of $\varLambda_{1} = 0{\kern 1pt} {\kern 1pt}$ and (*N* + 1) equations of $\varLambda_{j} = 0{\kern 1pt} {\kern 1pt} {\kern 1pt} {\kern 1pt} {\kern 1pt} (j = 2,3)$, we get 2 *N* + *M*+1 equations, i.e.

46 $$\begin{aligned} \varLambda_{ji} \approx 0,{\kern 1pt} \quad j = 1\quad i = 1, \ldots ,M - 1. \hfill \\ \varLambda_{ji} \approx 0,\quad j = 2,3,{\kern 1pt} \quad i = 0,1, \ldots ,N.{\kern 1pt} \hfill \\ \end{aligned}$$

Equation (42) together with Eq. (46) gives (*M* + 1)(*N* + 1) equations, which can be solved for *u*_*ij*_, (*i* = 0, 1, …, *M*, *j* = 0, 1, …, *N*). So the unknown function *u*(*x*, *t*) can be find out.

## Error analysis

In real problems, we often tend to solve some equations with unknown exact solutions. Hence, when we apply our method to these kinds of problems, it is necessary to introduce a process for estimating the error function (Chen et al. 2014).

We consider *e*_*n*_(*x*, *t*) = *u*(*x*, *t*) − *u*_*mn*_(*x*, *t*) as the error function of the approximate solution *u*_*mn*_(*x*, *t*) for *u*(*x*, *t*), where *u*(*x*, *t*) is the exact solution of Eq. (2)

Therefore, *u*_*n*_(*x*, *t*) satisfies the following problem

47 $$\frac{{\partial u_{mn} \left( {x,t} \right)}}{\partial t} + a\left( x \right)\frac{{\partial u_{mn} \left( {x,t} \right)}}{\partial x} = b\left( x \right)\frac{{\partial^{\nu } u_{mn} \left( {x,t} \right)}}{{\partial x^{\nu } }} + f\left( {x,t} \right) + R_{mn} \left( {x,t} \right)$$

The perturbation term *R*_*mn*_(*x*, *t*) can be obtained by substituting the estimated solution *u*_*mn*_(*x*, *t*) into the equations:

48 $$R_{mn} \left( {x,t} \right) = \frac{{\partial u_{mn} \left( {x,t} \right)}}{\partial t} + a\left( x \right)\frac{{\partial u_{mn} \left( {x,t} \right)}}{\partial x} - b\left( x \right)\frac{{\partial^{\nu } u_{mn} \left( {x,t} \right)}}{{\partial x^{\nu } }} - f\left( {x,t} \right)$$

Subtracting Eq. (47) from Eq. (2), we get the following equations:

49 $$\frac{{\partial e_{mn} \left( {x,t} \right)}}{\partial t} + a\left( x \right)\frac{{\partial e_{mn} \left( {x,t} \right)}}{\partial x} - b\left( x \right)\frac{{\partial^{\nu } e_{mn} \left( {x,t} \right)}}{{\partial x^{\nu } }} = - R_{mn} \left( {x,t} \right)$$

Obviously the above equation is one-dimensional fractional convection diffusion equation in which the error function *e*_*mn*_(*x*, *t*), is the unknown function. We can easily apply our method to the above equation to find an approximation of the error function $e^{\prime}_{mn} \left( {x,t} \right)$.

## Numerical simulation

In this section, we apply the proposed algorithm in the previous section to obtain numerical solutions of some convection diffusion equations with variable coefficients.

### *Example 1*

Consider the two-dimensional fractional convection diffusion equations with homogeneous initial-boundary conditions

50 $$\frac{{\partial u\left( {x,t} \right)}}{\partial t} + 2\frac{{\partial u\left( {x,t} \right)}}{\partial x} = \frac{{\partial^{1.5} u\left( {x,t} \right)}}{{\partial x^{1.5} }} + f\left( {x,t} \right){\kern 1pt} {\kern 1pt} {\kern 1pt} {\kern 1pt} {\kern 1pt} {\kern 1pt} {\kern 1pt} {\kern 1pt} {\kern 1pt} {\kern 1pt} {\kern 1pt} {\kern 1pt} {\kern 1pt} {\kern 1pt} {\kern 1pt} {\kern 1pt} {\kern 1pt} {\kern 1pt} {\kern 1pt} 0 < x < 1,t > 0$$

where $f(x,t) = x\left( {x - 1} \right)\left( {2t - 1} \right) + 2t\left( {t - 1} \right)\left( {2x - 1} \right) - {{4\sqrt x t\left( {t - 1} \right)} \mathord{\left/ {\vphantom {{4\sqrt x t\left( {t - 1} \right)} {\sqrt \pi }}} \right. \kern-0pt} {\sqrt \pi }}$, and the initial-boundary conditions:

$$\begin{aligned} u\left( {x,0} \right) &= 0,\\ u\left( {0,t} \right) &= u\left( {1,t} \right) = 0,\quad t > 0. \\ \end{aligned}$$

**Fig. 2:**
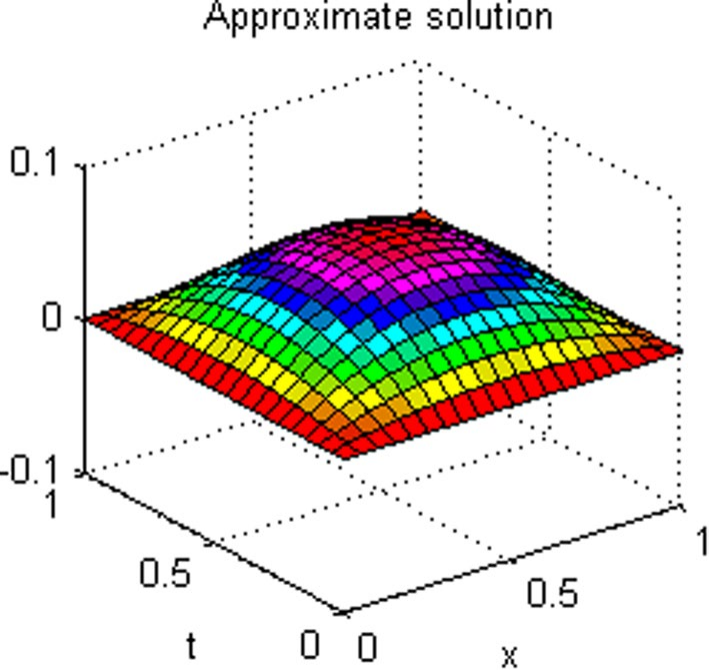
Approximate solution of Example 1

**Fig. 3:**
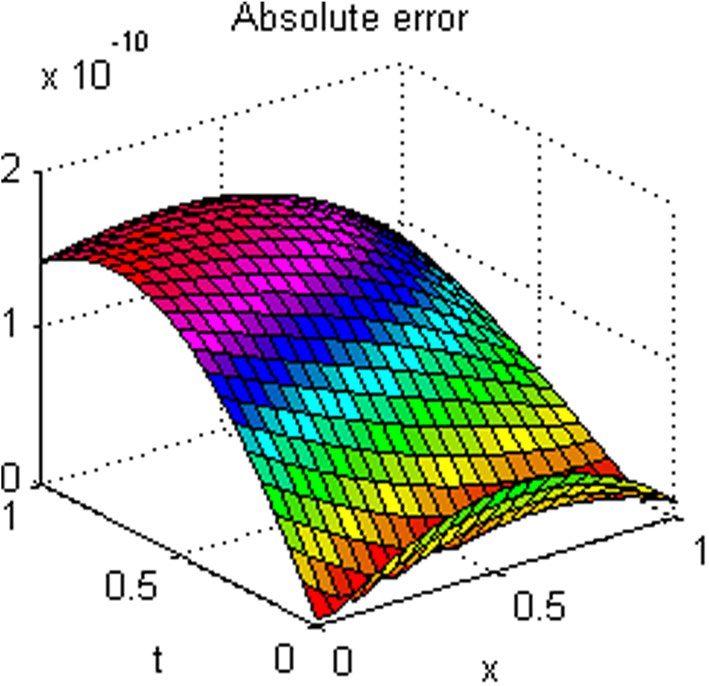
Absolute error of Example 1

**Fig. 4:**
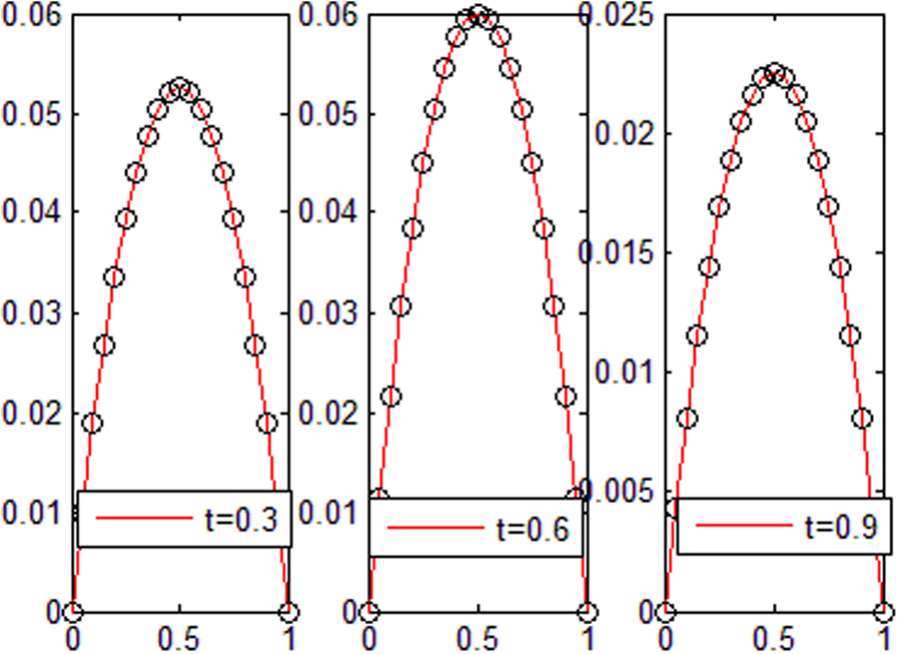
Numerical and exact solution in different values of *t* for Example 1

**Fig. 5:**
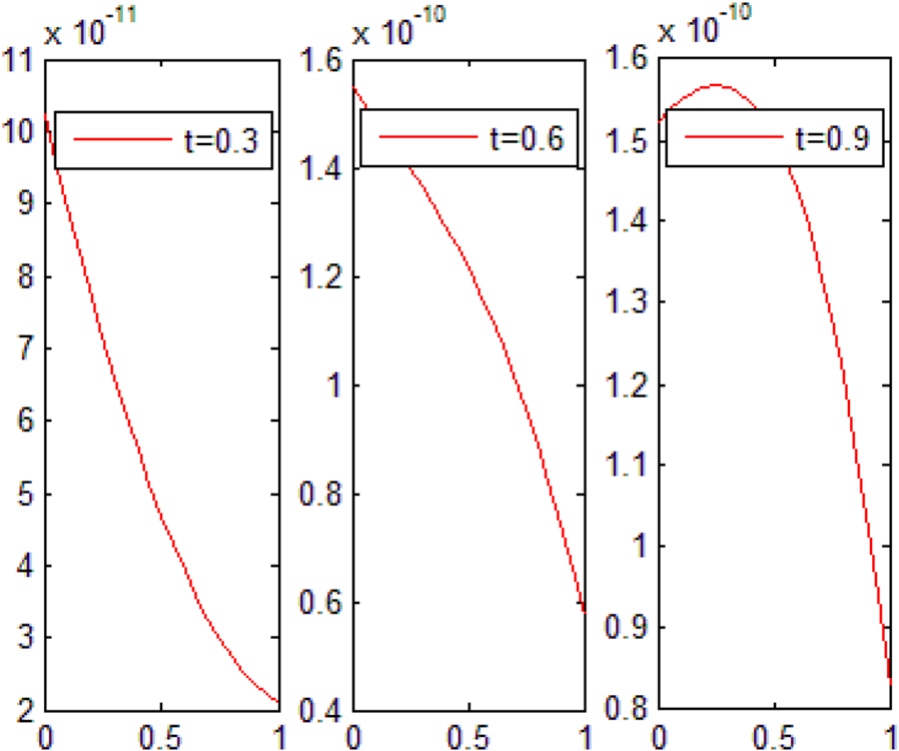
Absolute error in different values of *t* for Example 1

The exact solution of this problem is *u*(*x*, *t*) = *xt*(*x* − 1)(*t* − 1). The graphs of numerical solution for *M* = *N* = 4 is shown in Fig. 2. Absolute error between the numerical and analytical solutions is shown in Fig. 3. The graphs of analytical and approximate solutions for some nodes in (0, 1) × (0, 1) are presented in Fig. 4. Absolute error between the numerical and analytical solutions are also shown at different times in Fig. 5.

Figures 2 and 4 show that the numerical solutions are very close to the analytical solutions. Figures 3 and 5 show that the proposed algorithm has a high convergence precision.

### *Example 2*

**Fig. 6:**
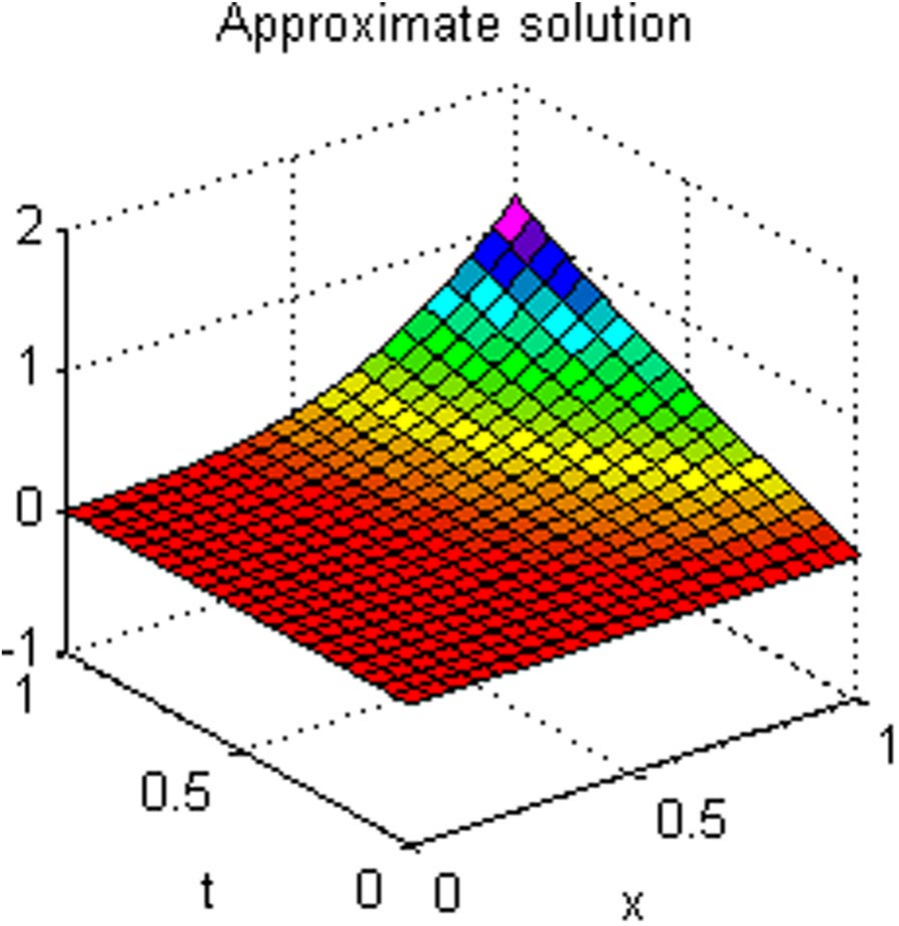
Approximate solution of Example 2

**Fig. 7:**
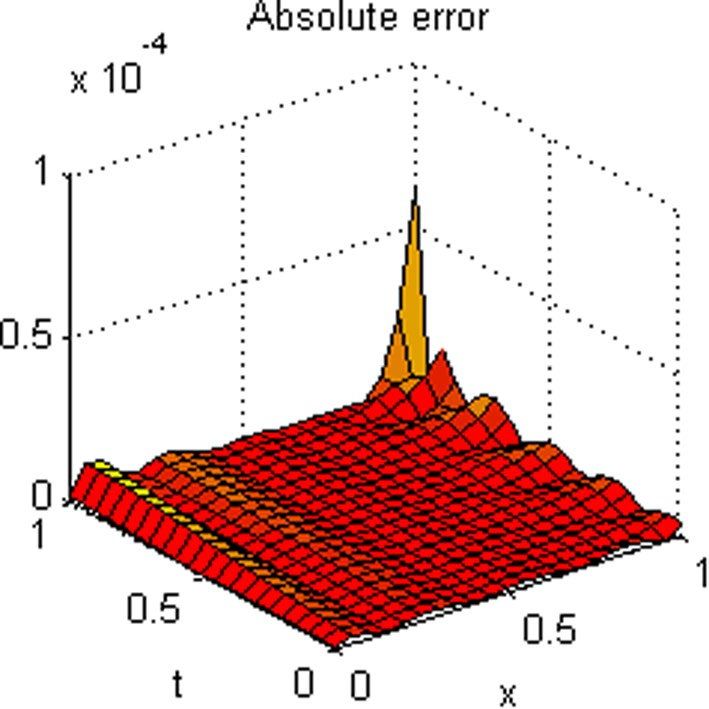
Absolute error of Example 2

**Fig. 8:**
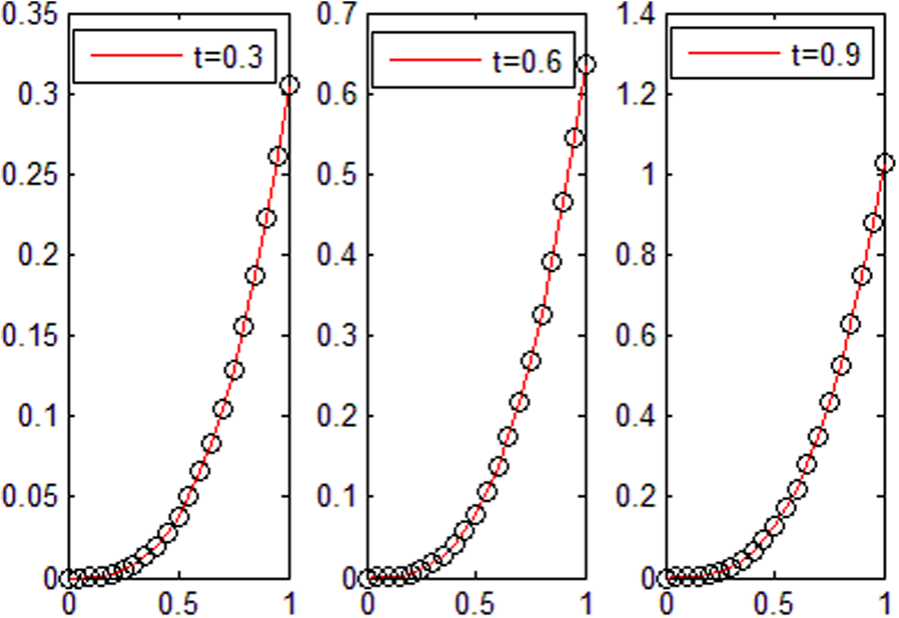
Numerical and exact solution in different values of *t* for Example 2

**Fig. 9:**
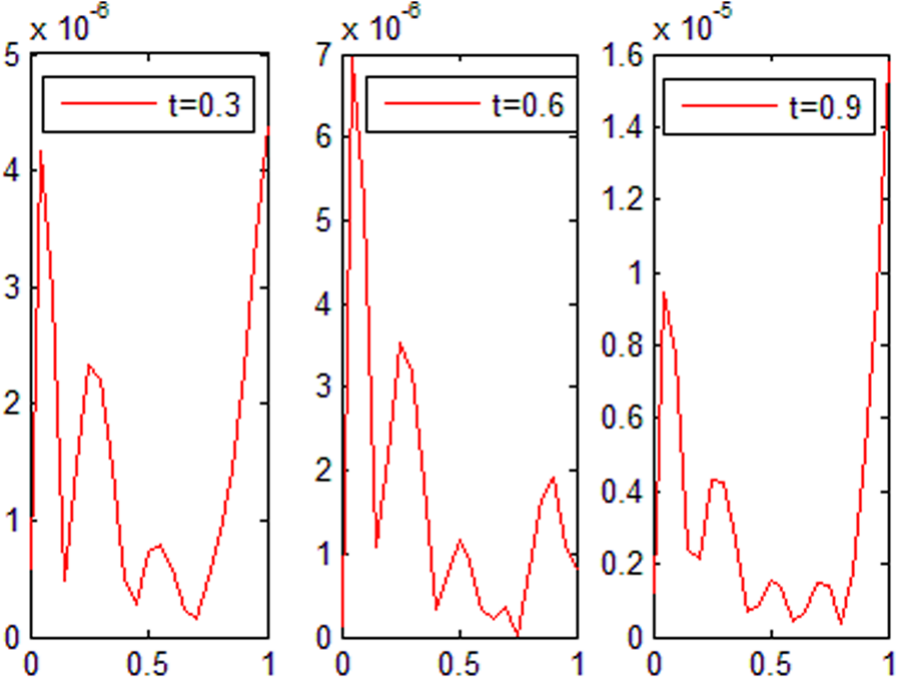
Absolute error in different values of *t* for Example 2

Consider the fractional convection diffusion equations with variable coefficients

51 $$\frac{\partial u}{\partial t} + a\left( x \right)\frac{\partial u}{\partial x} = b\left( x \right)\frac{{\partial^{2} u}}{{\partial x^{2} }} + f\left( {x,t} \right);{\kern 1pt} {\kern 1pt} {\kern 1pt} {\kern 1pt} {\kern 1pt} {\kern 1pt} {\kern 1pt} {\kern 1pt} {\kern 1pt} {\kern 1pt} 0 < x < 1,t > 0{\kern 1pt}$$

with the initial-boundary conditions:

$$\begin{aligned} u\left( {x,0} \right) &= 0, \\ u\left( {0,t} \right) &= 0,u(1,t) = \sinh (t),\quad t > 0 \\ \end{aligned}$$

where $a\left( x \right) = \frac{x}{3},{\kern 1pt} {\kern 1pt} {\kern 1pt} {\kern 1pt} {\kern 1pt} b\left( x \right) = \frac{{x^{2} }}{6};\,\,\,f\left( {x,t} \right) = x^{3} \cosh (t)$. The exact solution of this problem is $u\left( {x,t} \right) = x^{3} \sinh (t)$. The graph of the numerical solution for *M* = *N*=7 is shown in Fig. 6. Absolute error between the numerical and analytical solutions is shown in Fig. 7. The graphs of analytical and numerical solution for different *M* and *N* in some nodes are shown in Fig. 8. Absolute error between the numerical and analytical solution are also shown at different times in Fig. 9.

From Figs. 6 and 8, we can conclude that the numerical solutions converge to the exact solutions very well. Figures 7 and 9 show that the proposed algorithm can get a high convergence precision for one-dimensional convection diffusion equations with variable coefficients. Table 1 and Fig. 11 show that the convergence and accuracy of the proposed algorithm is very good, with *M* and *N* increase. Moreover, a small *M* and *N* can achieve high precision.

**Table 1:** Absolute error between approximate and exact solutions at *t* = 0.3 for Example 2

x	Exact solution	*M* = *N* = 3	*M* = *N* = 5	*M* = *N *= 7
0.1	0.0003	1.63e−005	3.06e−006	2.68e−006
0.2	0.0024	4.85e−005	2.08e−006	1.42e−006
0.3	0.0082	5.24e−005	2.60e−007	2.06e−006
0.4	0.0195	3.67e−005	9.20e−007	4.50e−007
0.5	0.0381	1.04e−005	9.70e−007	6.90e−007
0.6	0.0658	1.76e−005	1.10e−007	6.00e−007
0.7	0.1045	3.85e−005	1.10e−006	7.00e−008
0.8	0.1559	4.33e−005	1.98e−006	3.90e−007
0.9	0.2220	2.33e−005	2.14e−006	1.84e−006

Examples 2 and 3 show that the absolute error also can reaches to 10^−6^ for general one-dimensional fractional convection diffusion equations with variable coefficients. The two examples show that the proposed approach is very feasible and effective in solving fractional convection diffusion equations under real backgrounds.

### *Example 3*

Consider the convection diffusion equations with variable coefficients

52 $$b\left( x \right)\frac{\partial u}{\partial x} + \frac{\partial u}{\partial t} = a\left( x \right)\frac{{\partial^{\alpha } u}}{{\partial x^{\alpha } }} + g\left( {x,t} \right);{\kern 1pt} {\kern 1pt} {\kern 1pt} {\kern 1pt} {\kern 1pt} {\kern 1pt} {\kern 1pt} {\kern 1pt} {\kern 1pt} {\kern 1pt} 0 < x < 1,t > 0$$

where $a\left( x \right) = \frac{{\varGamma \left( {2.2} \right)}}{6}x^{2.8} ,{\kern 1pt} {\kern 1pt} {\kern 1pt} {\kern 1pt} {\kern 1pt} {\kern 1pt} b\left( x \right) = \frac{{x^{2} }}{3};$ with $g\left( {x,t} \right) = - x^{3} e^{ - t}$ and the boundary condition:

53 $$\begin{aligned} u\left( {x,0} \right) &= x^{3} \\ u\left( {0,t} \right) &= 0,\quad u\left( {1,t} \right) = e^{ - t} ,\qquad t > 0. \\ \end{aligned}$$

**Fig. 10:**
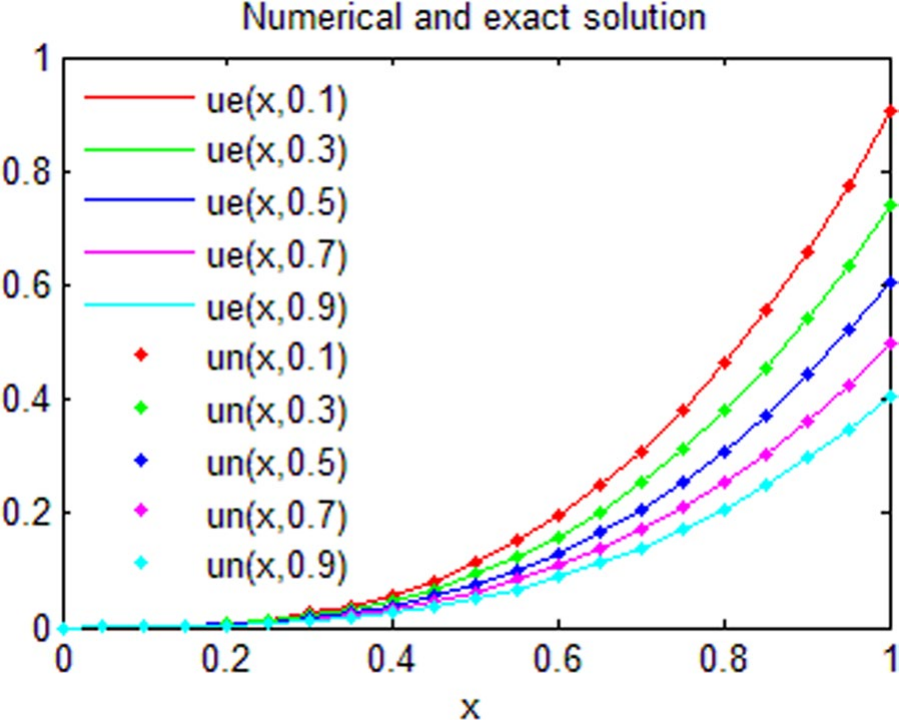
Comparison between numerical and analytical solution for Example 3

**Fig. 11:**
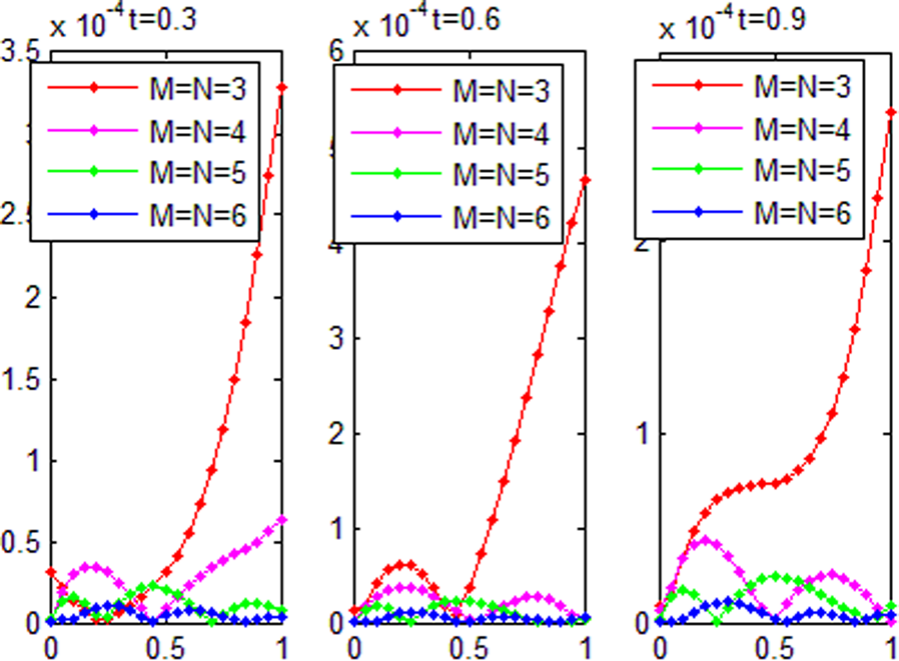
Absolute error for different *M* and *N* in some times for Example 3

The exact solution of this problem for *α* = 1.8 is $u\left( {x,t} \right) = x^{3} e^{ - t} .$ The graphs of comparison between numerical and analytical solution for *M* = *N* = 6 in some values of *t* are shown in Fig. 10. The graphs of absolute error for different *M* and *N* in some values of *t* are also shown in Fig. 11.

### *Example 4*

Consider the convection diffusion Eq. (52), with $$a(x) = \frac{{x^{2} }}{6},b(x) = \frac{x}{3}$$$$g\left( {x,t} \right) = - x^{3} e^{ - t}$$. The exact solution of this problem when *α* = 2 is $$u\left( {x,t} \right) = x^{3} e^{ - t}$$. The values of exact solution (*α* = 2) and approximate solution for some different values of *α* and some nodes (*x*, *t*) in (0, 1) × (0, 1), when *M* = *N* = 3 are shown in Table 2.

**Table 2:** Absolute error for different fractional order

(*x*, *t*)	*α* = 2	*α* = 1.95	*α* = 1.9	*α* = 1.85	*α* = 1.8
(0.2,0.2)	6.31e−05	0.0011	0.0025	0.0044	0.0066
(0.4,0.4)	7.45e−05	0.0006	0.0010	0.0011	0.0010
(0.6,0.6)	1.64e−04	0.0034	0.0069	0.0109	0.0154
(0.8,0.8)	5.81e−05	0.0040	0.0085	0.0140	0.0205

By comparing the data in Table 2, we can see the numerical solutions agree with the analytical solution (*α* = 2) well with the fractional order gradually approximate to the order of *α* = 2. The example is introduced to verify the stability of the proposed algorithm.

## Conclusion

Here a new operational method to approximate the numerical solution of one-dimensional fractional convection diffusion equations have been introduced. To this end, the new operational matrix of fractional-order differentiation is obtained. It appears that using Chebyshev operational matrix algorithm will give more accurate solutions than other existing methods. The approach is computationally efficient and the algorithm can be implemented easily on a computer. The advantage of the methods is that only small size operational matrix is required to provide the solutions at high accuracy. Numerical examples are given to show that the proposed algorithm is robust, efficient and applicable.
